# Targeting Foodborne Pathogens with Bacteriophages: Mechanisms, Applications, and Resistance

**DOI:** 10.3390/pathogens15070711

**Published:** 2026-07-07

**Authors:** Lekshmi K. Edison, Subhashinie Kariyawasam

**Affiliations:** Department of Comparative, Diagnostic, and Population Medicine, College of Veterinary Medicine, University of Florida, Gainesville, FL 32610, USA; skariyawasam@ufl.edu

**Keywords:** bacteriophages, foodborne pathogens, phage biocontrol, One Health, *Salmonella*, *Campylobacter*, *Listeria monocytogenes*, Shiga toxin-producing *Escherichia coli*, antimicrobial resistance, biofilms, food safety

## Abstract

Foodborne pathogens remain a major public health challenge, particularly in the context of antimicrobial resistance and persistent contamination across animal, food-processing, and retail environments. This review examines bacteriophages as precision antimicrobials for controlling major foodborne bacteria, including *Salmonella*, *Campylobacter*, Shiga toxin-producing *Escherichia coli* (STEC), *Listeria monocytogenes*, and *Vibrio* spp., and summarizes the biological basis of phage-mediated control: strictly lytic life cycles, receptor-specific adsorption, direct bacterial killing, biofilm disruption, and resistance-associated fitness trade-offs. It further discusses pre-harvest, post-harvest, and processing-environment applications, with emphasis on matrix-dependent efficacy, delivery strategies, commercial products, and regulatory status. While bacteriophages offer high specificity and may help preserve the native microbiome, their integration into multi-hurdle food-safety systems require careful validation because their performance is influenced by narrow host ranges, bacterial resistance, food-matrix effects, formulation constraints, and regulatory complexity and scale-up challenges. Broader implementation will require rationally designed phage-cocktails, thorough genomic safety screening, matrix-specific validation studies, scalable manufacturing processes, and continuous monitoring for post-application resistance. Overall, bacteriophages should be viewed as promising but context-dependent adjuncts to validated food-safety and One Health frameworks, rather than stand-alone solution for reducing foodborne pathogen burdens.

## 1. Introduction

Foodborne diseases continue to constitute a significant global public health and economic burden. The latest World Health Organization (WHO) estimates of the global burden of foodborne diseases, released in 2026, indicate that unsafe food causes approximately 866 million illnesses and 1.5 million deaths annually worldwide. These updated estimates cover 42 major foodborne hazards across 194 countries from 2000 to 2021. Children under five years of age account for a disproportionate share of the foodborne disease burden, and low- and middle-income countries, particularly in Africa and Southeast Asia, continue to experience the highest impact. Unsafe food also causes substantial economic losses, with global productivity losses estimated at approximately US$310 billion in 2021 [1]. More than 200 distinct foodborne diseases have been identified [2], caused by a wide range of agents, including bacterial pathogens (e.g., *Salmonella*, *Campylobacter*, *Escherichia coli*, *Listeria monocytogenes*, and *Staphylococcus aureus*), viruses (e.g., noroviruses, hepatitis viruses, and rotaviruses), parasites, and various toxins [2,3]. Recent surveillance data further demonstrate that bacterial foodborne pathogens remain difficult to control despite modern food-safety systems. In the 2024 European Union (EU) One Health Zoonoses Report, campylobacteriosis and salmonellosis were the first and second most frequently reported zoonoses in humans, followed by Shiga toxin-producing *Escherichia coli* (STEC) infections. Listeriosis was less common but remained the most severe zoonotic disease, with the highest percentage of hospitalizations and the highest case-fatality rate. The same report documented increasing five-year trends for campylobacteriosis, salmonellosis, listeriosis, and STEC infections, underscoring the continued public health relevance of these pathogens [4]. The global burden of foodborne disease is not uniformly distributed. It disproportionately affects low- and middle-income countries, particularly regions in Africa and Southeast Asia, as well as vulnerable populations such as children under five years of age, who experience the highest incidence and mortality rates. Estimates from 2010 indicate that bacterial diarrheal pathogens, including non-typhoidal *Salmonella* and *Campylobacter* species, were responsible for hundreds of millions of cases and accounted for millions of disability-adjusted life years (DALYs) worldwide [5].

Current control strategies, including improved hygienic practices, on-farm biosecurity, pathogen surveillance and vaccination, and advancements in food processing, have contributed substantially to reducing the burden of foodborne diseases. However, these measures remain insufficient [6,7]. For example, *Campylobacter* remains difficult to control in poultry production because intestinal colonization can persist despite farm-level biosecurity and processing interventions [8,9]. *Salmonella* continues to cause outbreaks linked to poultry, eggs, meat, fresh produce, and other food vehicles [10]. Effective vaccines are available for only a limited number of pathogens and are not universally implemented [11]; vaccine options remain limited or impractical for several major foodborne bacteria, including *Campylobacter*, STEC, and *L. monocytogenes*. The application of chemical sanitizers and antibiotics may adversely affect food quality and disrupt the host microbiome [12,13]. In addition, sanitizer efficacy can be reduced by organic matter, surface irregularities, and mature biofilms, which can protect pathogens on equipment and food-contact surfaces [14,15]. Furthermore, the extensive use of antibiotics in both agriculture and clinical settings has accelerated the emergence and dissemination of antimicrobial resistance (AMR) among foodborne pathogens. The WHO has identified AMR as a critical and escalating global health threat, with projections suggesting it may soon become a leading cause of mortality worldwide [16,17]. Because antibiotics are not suitable as routine food-safety interventions, these limitations highlight the need for complementary, targeted, and sustainable antimicrobial strategies that can be integrated into existing food-safety systems. In this context, there is an urgent need to develop alternative and sustainable antimicrobial strategies. Bacteriophages, the most abundant biological entities on earth, have re-emerged as promising precision antimicrobials. These viruses specifically infect bacterial hosts by recognizing surface receptors and, during the lytic cycle, replicate within and ultimately lyse the target cell. This remarkable host specificity confers a key advantage, as phage-based interventions can selectively target pathogenic bacteria without disrupting beneficial microbiota, thereby preserving microbial homeostasis [18,19,20].

Regulatory acceptance of phage-based interventions has expanded substantially over the past two decades, particularly in the United States, where the Food and Drug Administration (FDA) has granted “Generally Recognized as Safe” (GRAS) status to several phage preparations targeting foodborne pathogens such as *L. monocytogenes*, *Salmonella*, and *E. coli* and has approved their application in ready-to-eat foods, meats, dairy products, and fresh produce [21,22]. Notably, bacteriophage P100 (Listex™ P100) has been extensively evaluated for safety and efficacy, with both FDA approvals and European Food Safety Authority (EFSA) assessments indicating that its use does not pose significant risks to human health and can effectively reduce *Listeria* contamination in food products [23,24]. Beyond regulatory validation, commercial and near-commercial applications of bacteriophages now span diverse food-processing and agricultural contexts, including surface decontamination of meats, dairy products, and fresh produce, as well as biocontrol strategies in crop production and livestock systems, with multiple products approved or in development under the FDA, United States Department of Agriculture (USDA), and United States Environmental Protection Agency (EPA) frameworks [25]. However, regulatory pathways for phage-based food applications differ substantially across countries and regions, where phage products may be evaluated as food additives, processing aids, feed additives, veterinary products, plant-protection agents, or biocontrol products depending on the intended use, food matrix, application stage, and national regulatory framework. In addition to whole-phage applications, phage-derived enzymes, such as endolysins and depolymerases, are emerging as potent antimicrobial agents capable of degrading bacterial cell walls and biofilm matrices while reducing concerns about horizontal gene transfer. However, their deployment still requires rigorous safety and efficacy validation [26]. Despite these advantages, important limitations must be considered for successful implementation: (i) the inherently narrow host range of phages necessitates the use of carefully designed phage cocktails; (ii) the efficacy can be strongly influenced by environmental and food matrix conditions, including pH, temperature, moisture, and surface characteristics; and (iii) the evolutionary capacity of bacteria to develop phage resistance requires proactive mitigation strategies such as phage rotation or combination therapies [18,27,28]. High specificity, regulatory acceptance, rising commercial use, and rapid biotechnological progress underscore the growing importance of bacteriophages as precision antimicrobials in modern food safety and One Health frameworks.

The urgency of advancing alternative antimicrobial strategies in food systems is highlighted by persistent epidemiological trends indicating that major foodborne pathogens remain inadequately controlled despite modern interventions. Recent European surveillance data show that *Campylobacter* and *Salmonella* continue to be the most frequently reported bacterial causes of gastroenteritis, while *L. monocytogenes*, although less common, accounts for a disproportionately high burden of hospitalizations and mortality. Notably, the most recent EU One Health Zoonoses Report documented that listeriosis incidence has reached its highest level since 2007, with increasing case numbers and severe clinical outcomes, particularly among vulnerable populations [29,30]. In parallel, STEC continues to pose significant outbreak potential and long-term sequelae [31]. Compounding these foodborne pathogen threats, AMR is increasingly compromising treatment efficacy and complicating control strategies across interconnected human, animal, food-production, and environmental systems, thereby amplifying the need for sustainable and targeted interventions. Against these converging pressures, this review aims to critically evaluate whether and how phage-based interventions, either as standalone approaches or integrated within multi-hurdle systems for food safety, can achieve measurable, scalable, and regulatorily acceptable reductions in the pathogen burden while minimizing unintended ecological impacts and preserving microbiome integrity.

From a One Health perspective, phage-based food-safety interventions should be evaluated across the full continuum linking animal reservoirs, food-production environments, environmental dissemination, and human exposure [32,33]. Foodborne pathogens such as *Salmonella*, *Campylobacter*, STEC, and *L. monocytogenes* can persist in livestock, poultry, manure, water, soil, processing environments, biofilms, and food products, creating multiple opportunities for transmission to humans [4]. Accordingly, phage applications may contribute to One Health goals by reducing pathogen carriage in food animals, limiting contamination during processing, lowering environmental persistence on food-contact surfaces, and potentially decreasing reliance on conventional antimicrobials. However, One Health implementation also requires monitoring for phage resistance, unintended microbiome effects, environmental persistence of applied phages, and possible impacts on AMR dynamics [33,34]. Therefore, phage biocontrol should be viewed as a targeted intervention within integrated farm-to-fork surveillance and control systems rather than as an isolated food-processing tool.

Although numerous reviews have described bacteriophage biology, phage–host interactions, or selected applications of phages against foodborne pathogens, the available literature remains fragmented across pathogen groups, food matrices, production stages, and regulatory contexts. Many existing summaries emphasize the antimicrobial potential of phages, but only a few critically integrate how efficacy varies across pathogen species or strains, food matrix composition, application route, contact time, storage conditions, resistance development, formulation stability, and commercial feasibility. This creates an important knowledge gap for translating promising laboratory findings into predictable, scalable, and regulatorily acceptable food-safety interventions. Therefore, this review differs from previous summaries by evaluating bacteriophages not simply as antibacterial agents, but as context-dependent precision tools within pre-harvest, post-harvest, processing-environment, and One Health food-safety systems. Particular emphasis is placed on matrix-dependent efficacy, stage-specific delivery strategies, biofilm control, resistance-associated fitness trade-offs, mitigation approaches, commercial products, regulatory considerations, and implementation barriers. By integrating mechanistic, applied, regulatory, and translational perspectives, this review aims to clarify where phage-based interventions are most promising, where their limitations remain substantial, and what research priorities are required for their broader adoption in food-production systems. Thus, the unique contribution of this review is its integrated evaluation of phage-based food-safety interventions across biological mechanisms, matrix-dependent efficacy, resistance management, regulatory readiness, and real-world implementation challenges.

### 1.1. Timeline of Milestones in Phage Applications for Food Safety

**Figure 1:**
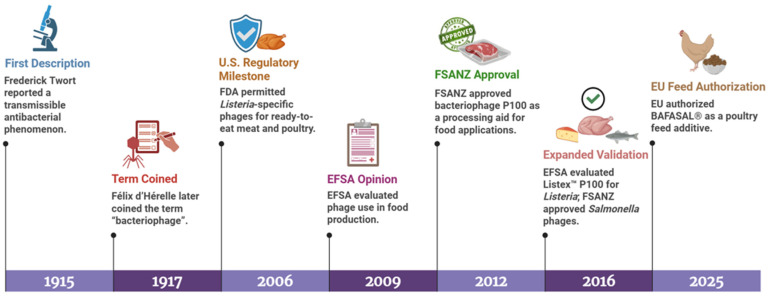
Timeline of key milestones in bacteriophage applications for food safety (Created in https://BioRender.com, accessed on 6 May 2026). Abbreviations: FDA, United States Food and Drug Administration; EFSA, European Food Safety Authority; FSANZ, Food Standards Australia New Zealand; EU, European Union; BAFASAL^®^, bacteriophage-based feed additive targeting poultry-associated *Salmonella*.

The historical development of bacteriophage applications in food safety reflects a gradual transition from discovery to regulated implementation. Bacteriophages were first described by Frederick Twort in 1915, and Félix d’Hérelle later coined the term “bacteriophage” in 1917. Their use for controlling foodborne pathogens gained prominence in the contemporary regulatory landscape. A key milestone occurred in 2006, when the FDA permitted a *L. monocytogenes*-specific bacteriophage preparation for ready-to-eat meat and poultry products and issued a GRAS “no questions” response for the use of the bacteriophage P100 in cheeses. Subsequent developments included EFSA’s 2009 scientific opinion on bacteriophages in food production, Food Standards Australia New Zealand (FSANZ)’s approval of P100 as a processing aid in 2012, EFSA’s 2016 evaluation of Listex™ P100 for ready-to-eat foods, FSANZ’s approval of *Salmonella* phage processing aids in 2016, and the EU’s authorization of BAFASAL^®^ as a bacteriophage feed additive for poultry in 2025. Together, these milestones demonstrate the transition of phages from experimental antibacterial agents to regulated tools for targeted pathogen control in food-processing, feed, and One Health food-safety systems. A summary of these key historical and regulatory milestones is presented in Figure 1.

### 1.2. Literature Search and Selection Strategy

This review was prepared as a narrative review rather than a systematic review or meta-analysis. Relevant literature was identified through searches of PubMed, Web of Science, Scopus, and Google Scholar using combinations of keywords including “bacteriophage”, “phage biocontrol”, “foodborne pathogens”, “food safety”, “*Salmonella*”, “*Campylobacter*”, “*Listeria monocytogenes*”, “Shiga toxin-producing *Escherichia coli*”, “*Vibrio*”, “biofilm”, “phage resistance”, “phage cocktail”, “pre-harvest”, “post-harvest”, “food processing”, “regulatory approval”, and “commercial phage products.” Priority was given to peer-reviewed research articles, review articles, regulatory documents, and safety assessments relevant to phage applications in food production and food-processing systems. Studies were selected based on their relevance to phage mechanisms, food-matrix efficacy, biofilm control, resistance development, commercial translation, regulatory status, and One Health implications. Older foundational studies were included where necessary to provide historical or mechanistic context, while recent publications were prioritized to reflect current developments in phage-based food-safety applications.

## 2. Biology of Bacteriophages Relevant to Food Safety

For food-safety applications, the relevance of bacteriophage biology is determined less by general phage classification and more by how specific biological traits influence safety, efficacy, and practical performance in food-production systems. Key determinants include life-cycle strategy, receptor specificity, adsorption efficiency, replication kinetics, host range, genomic safety, environmental stability, and compatibility with the intended food matrix. These features directly affect whether a phage can produce reliable pathogen reduction under realistic pre-harvest, post-harvest, processing, or storage conditions. Therefore, phages intended for food biocontrol should be evaluated not only for antibacterial activity in laboratory culture but also for their performance under matrix-specific and process-relevant conditions.

### 2.1. Preference for Strictly Lytic Phages in Food Biocontrol

**Figure 2:**
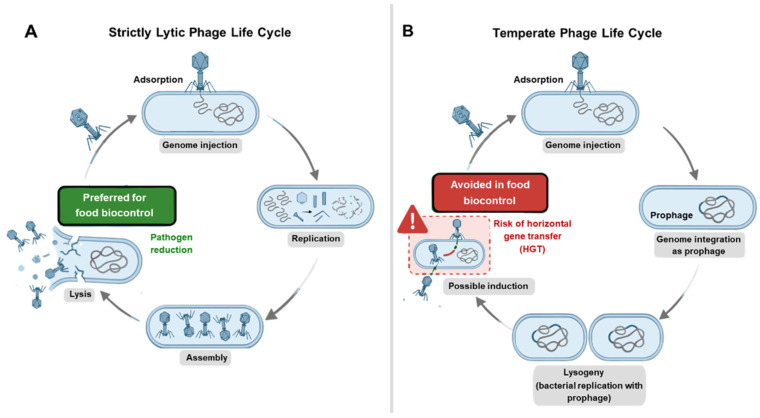
Comparison of strictly lytic and temperate bacteriophage life cycles and their relevance to food biocontrol. (**A**) Strictly lytic phages reduce bacterial populations through adsorption, genome injection, intracellular replication, assembly, and host-cell lysis, and are therefore preferred for food biocontrol. (**B**) Temperate phages can establish lysogeny through prophage formation and may later be induced into the lytic cycle. Because this pathway may promote horizontal gene transfer (HGT), including the dissemination of virulence and antimicrobial resistance (AMR) determinants, temperate phages are generally avoided in food safety applications (Created in https://BioRender.com, accessed on 6 May 2026).

Strictly lytic phages are preferred for food biocontrol because they infect and lyse bacterial hosts without establishing lysogeny. Their infection cycle involves adsorption to bacterial surface receptors, genome injection, intracellular replication, assembly of progeny virions, and host-cell lysis, resulting in direct bacterial inactivation. In contrast, temperate phages can integrate into the bacterial host genome as a prophage and may contribute to horizontal gene transfer (HGT). This raises safety concerns because temperate phages may carry or mobilize genes associated with virulence, toxins, lysogeny, or AMR. For this reason, temperate phages are generally unsuitable for food-safety applications, whereas strictly lytic phages provide a more predictable and controllable antimicrobial strategy (Figure 2) [18,35].

This distinction has practical regulatory importance. Regulatory agencies generally require that bacteriophages used for food biocontrol be strictly lytic, thoroughly characterized, and free of genes associated with lysogeny, toxins, virulence, or AMR. For example, the FDA regulation for *L. monocytogenes*-specific bacteriophage preparations defines the approved food additive as “lytic-type” bacteriophages lacking lysogenic activity, thereby incorporating the absence of lysogeny as an essential safety criterion for food-use phage products [36]. Similarly, GRAS dossiers and European risk assessments emphasize genomic characterization, host specificity, the absence of undesirable genetic elements, and controlled manufacturing quality as central components of phage safety evaluation [37,38]. Genomic safety screening generally involves whole-genome sequencing, genome annotation, and bioinformatic analysis to screen lysogeny-associated genes, toxin or virulence genes, AMR determinants, and genes associated with HGT or transduction [39]. However, the use of strictly lytic phages alone does not guarantee effective pathogen control. A phage that is genomically safe may still perform poorly if it has a narrow host range, weak adsorption under food-relevant conditions, limited stability during storage, or poor access to bacterial cells within complex matrices or biofilms. Therefore, safety and efficacy must be assessed together. The most suitable food-use phages are those that combine lytic activity, genomic safety, environmental stability, and demonstrated performance in the intended food system.

Another key feature supporting the use of bacteriophages in food safety is their high host specificity. Unlike broad-spectrum chemical sanitizers or antibiotics, phages typically infect a limited range of bacterial hosts, often at the species, serovar, or strain level. This specificity is primarily determined by the interaction between phage receptor-binding proteins and bacterial surface receptors [35,40]. In food biocontrol, this targeted activity is advantageous because it enables selective reduction of pathogens such as *Salmonella*, *Campylobacter*, *L. monocytogenes*, or STEC while sparing desirable background microbiota, including fermentative or other beneficial bacterial communities.

However, the host-specific nature of bacteriophages presents practical challenges. A narrow host range may limit the effectiveness of a single phage against genetically diverse pathogen populations encountered in food animals, raw ingredients, processing environments, and finished food products. For this reason, phage cocktails containing multiple well-characterized lytic phages are frequently used to broaden antibacterial coverage, reduce the likelihood of bacterial escape mutants, and improve efficacy across heterogeneous food matrices [18,41]. The design of such cocktails requires careful evaluation of host range, receptor usage, genomic safety, stability under processing conditions, and compatibility among phages. This compatibility testing is important because phages within a cocktail may not always act additively; potential antagonistic interactions can occur through competition for the same bacterial receptors or host cells, differences in adsorption and replication kinetics, or selection of shared resistance phenotypes, which may reduce overall biocontrol efficacy [42,43]. Importantly, combining phages that recognize different bacterial receptors may reduce the probability of resistance development, because bacterial escape would require simultaneous modifications or the loss of multiple surface structures, which might impose fitness costs on the pathogen [35].

### 2.2. Phage–Host Interaction Mechanisms

The antibacterial activity of bacteriophages in food systems begins with receptor-specific adsorption to the bacterial surface. This initial interaction determines host range and is one of the most important factors influencing phage efficacy. In Gram-negative foodborne pathogens, such as nontyphoidal *Salmonella*, *E. coli*, and *C. jejuni*, commonly recognized receptors include lipopolysaccharide (LPS), outer membrane proteins (OMPs), flagella, capsules, and pili. In Gram-positive bacteria such as *L. monocytogenes* and *Staphylococcus aureus*, phages may recognize cell wall teichoic acids, surface proteins, or other cell-wall-associated structures [18,28,44]. Receptor expressions can vary depending on bacterial strain, growth phase, temperature, nutrient availability, stress exposure, and food-matrix conditions; consequently, phage adsorption efficiency may differ between laboratory media and real food environments. Following adsorption, the phage injects its genome into the bacterial cell and redirects host metabolic machinery toward phage replication. During this intracellular phase, phage genes involved in genome replication, structural protein synthesis, assembly, and host-cell lysis are coordinately expressed. In strictly lytic phages, this process culminates in the production of progeny virions and the destruction of the bacterial cell [28,44]. The efficiency of this process is often described using parameters such as the latent period and the burst size [45,46]. The latent period is the time between phage adsorption and host-cell lysis, whereas burst size is the number of progeny phages released per infected bacterial cell [47,48]. These parameters are highly relevant for food biocontrol because they influence how rapidly phage populations can amplify and suppress bacterial contamination within a given food matrix [45].

Bacterial lysis is typically mediated by phage-encoded lytic enzymes, including endolysins and holins. Holins form lethal lesions in the bacterial cytoplasmic membrane, allowing endolysins to access and degrade the peptidoglycan layer, ultimately leading to cell rupture and release of progeny phages. In Gram-negative bacteria, additional proteins such as spanins may be required to disrupt the outer membrane during the final stage of lysis. This lytic mechanism directly contributes to pathogen reduction in foods and on food-contact surfaces [49,50,51]. However, the magnitude of bacterial reduction depends on several factors, including initial bacterial load, phage concentration, multiplicity of infection, adsorption efficiency, temperature, pH, water activity, food composition, surface structure, and the presence of organic matter. Food matrices can strongly influence phage–host interactions. Liquid foods may facilitate phage diffusion and bacterial contact, whereas solid or semi-solid foods can restrict phage movement and reduce adsorption efficiency. Fats, proteins, carbohydrates, salts, and natural antimicrobial compounds may also affect phage stability and access to bacterial cells. For example, phage performance on meat, dairy, fresh produce, and seafood may differ because each matrix presents distinct physicochemical barriers [52,53]. Therefore, phage efficacy observed under controlled laboratory conditions must be validated in the intended food system, using realistic processing, storage, and contamination conditions. Overall, the success of bacteriophage-based food biocontrol depends on the coordinated relationship between phage biology, bacterial receptor availability, replication kinetics, and food-matrix characteristics.

## 3. Mechanisms of Phage-Mediated Control of Foodborne Pathogens

**Figure 3:**
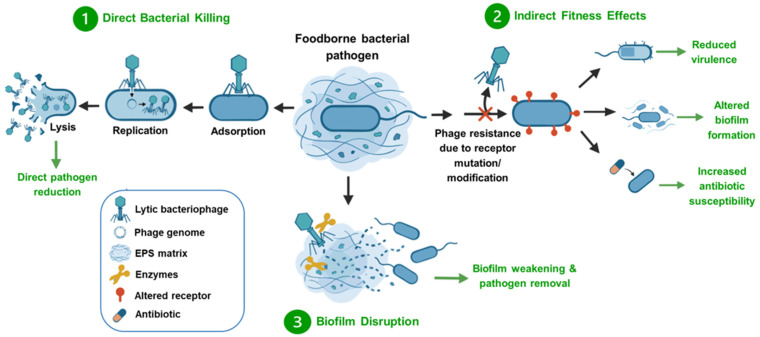
Mechanisms of phage-mediated pathogen control. Bacteriophages can reduce foodborne bacterial pathogens through three major mechanisms: direct bacterial killing, indirect fitness effects, and biofilm disruption. Direct killing occurs through phage adsorption, replication, and host-cell lysis, thereby reducing the pathogen load. Phage resistance caused by mutations or modifications in bacterial receptors may generate fitness trade-offs, including reduced virulence, altered biofilm formation, and increased antibiotic susceptibility. Phages and phage-derived enzymes (depolymerases or endolysins) can also weaken biofilms by penetrating the extracellular polymeric substance (EPS) matrix and degrading matrix components, thereby improving pathogen removal (Created in https://BioRender.com, accessed on 9 May 2026).

Bacteriophages reduce foodborne pathogens through multiple interconnected mechanisms rather than solely through direct bacterial lysis. As summarized in Figure 3, phage-mediated control can occur through three major pathways: direct bacterial killing, biofilm disruption, and indirect fitness effects. Together, these mechanisms indicate that phage-based biocontrol should be evaluated not only by short-term pathogen reduction, but also by its effects on biofilm persistence, bacterial fitness, and resistance-associated trade-offs.

### 3.1. Direct Bacterial Killing

The core bactericidal mechanism of a strictly lytic phage is governed by a coordinated intracellular infection sequence, such as adsorption to a surface receptor, genome injection, host takeover, phage genome replication, virion assembly, and programmed lysis. In Gram-negative hosts, lysis is typically organized as a staged holin–endolysin–spanin cascade, as mentioned previously. Holins permeabilize the inner membrane, allowing endolysins to access and hydrolyse the peptidoglycan layer, whereas spanins complete the final disruption of the outer membrane. Endolysins themselves comprise several catalytic classes, including muramidases, glucosaminidases, amidases, endopeptidases, and transglycosylases, which cleave specific bonds within the bacterial cell wall. Notably, when used as purified proteins, endolysins bypass phage replication entirely and act as stand-alone peptidoglycan hydrolases [54].

Although the latent period and burst size are important indicators of phage replication efficiency, they do not fully predict antibacterial performance in food systems. Short latent periods and large burst sizes can support rapid phage amplification. However, food matrices often modify this relationship by influencing phage diffusion, bacterial accessibility, host metabolism, and environmental stability. For example, refrigeration may suppress bacterial metabolic activity and limit phage replication, making pathogen reduction more strongly dependent on initial phage dose, surface coverage, and phage–host contact. Several studies illustrate this matrix-dependent effect. The *Salmonella* phage LPSE1 showed rapid infection kinetics and a burst size of approximately 94 phage-forming units (PFU) per infected cell, yet it performed poorly in milk at 4 °C. At 28 °C, however, *Salmonella* Enteritidis counts were lowered by 1.44–2.37 log_10_ CFU/mL [55]. Similarly, the lytic *Listeria* phage vB_LmoP_M15, despite a short latent period of 15–20 min and a large burst size of 172 PFU per infected cell, mainly suppressed bacterial growth in pasteurized milk without completely eliminating the pathogen [56]. These findings indicate that phage-mediated killing in foods depends not only on intrinsic phage biology but also on the physicochemical conditions of the target matrix.

Commercial and experimental studies further show that phage treatment generally produces meaningful direct pathogen killing. For example, the *Listeria* phage P100 reduced *L. monocytogenes* by approximately 1.8–3.5 log_10_ CFU/g on raw salmon fillets and by 2.1–2.3 log units in soft cheeses [57]. Similarly, *Salmonella*, STEC, and *Vibrio parahaemolyticus* phages have produced variable but significant reductions across chicken, milk, lettuce, beef, and seafood, depending on storage temperature, food structure, and phage accessibility [58,59,60,61]. Therefore, direct bacterial killing should be viewed as a matrix-dependent risk-reduction strategy, where phage selection must integrate replication kinetics with validation under realistic food-processing and storage conditions [55]. To provide a more systematic comparison of phage efficacy across different foodborne pathogen groups, food matrices, and application conditions, Table 1 summarizes representative food biocontrol phages, including their replication kinetics and food-matrix efficacy across pathogens such as *L. monocytogenes*, *Salmonella*, *E. coli*, and *V. parahaemolyticus*. The examples show that phage performance varies widely among pathogens and food systems, with reported reductions ranging from modest attenuation to several-log decreases depending on the target pathogen, food matrix, storage temperature, phage dose, contact time, surface structure, and initial contamination level.

### 3.2. Biofilm Disruption

Bacteriophages can also disrupt bacterial biofilms, which are important reservoirs of foodborne pathogens on processing equipment, food-contact surfaces, and raw commodities [64]. Phage-mediated biofilm control occurs through both infection-dependent and enzyme-dependent mechanisms [65,66]. In the infection-dependent pathway, phages infect exposed cells at the biofilm surface, and subsequent lysis releases progeny phages that can penetrate local voids and promote further rounds of infection. In parallel, some phages encode depolymerases associated with tail fibers or tail spikes, which degrade capsules, extracellular polymeric substances, or O-polysaccharides, thereby improving access to embedded bacterial cells [67]. Evidence from food-contact surfaces shows that phages can reduce biofilm biomass and viable bacterial contamination, although efficacy depends on strain, phage dose, surface type, and biofilm maturity. For example, a *Salmonella* Enteritidis phage cocktail reduced biofilm biomass on stainless-steel washers by 54–98%, while a 21-phage STEC cocktail produced immediate reductions on high-density polyethylene and stainless-steel surfaces [68,69]. Surface composition is also important, as phage phT4A reduced *E. coli* biofilms more effectively on plastic than on stainless steel [70].

Phage applications are particularly relevant to food-processing environments, where persistent biofilms can serve as sources of recurrent contamination. A phage cocktail reduced *E. coli* O157:H7 biofilm-associated contamination on spinach-harvester blades by 4.5 log_10_ CFU per blade after 2 h, while the *L. monocytogenes* phage vB_LmoP_M15 disrupted preformed biofilms and inhibited the formation of new biofilms [56,71]. However, mature or matrix-rich biofilms can limit phage diffusion and receptor access, leading to incomplete biofilm removal or partial rebound after treatment. Therefore, phages may be most effective as part of multi-hurdle sanitation strategies. For instance, combining phage treatment with cold nitrogen plasma achieved greater reductions in *E. coli* O157:H7 biofilms than either intervention alone, supporting the role of phages as targeted biosanitation tools within broader food-processing hygiene programs [69,72].

In addition to whole-phage applications, phage-derived enzymes offer targeted approaches for disrupting bacterial cell walls, capsules, O-antigens, and biofilm-associated polysaccharides. Depolymerases provide an additional strategy by weakening biofilm structure. For example, the *E. coli* O157-specific depolymerase Dpo10, an engineered bacteriophage-derived enzyme, inhibited biofilm formation without directly suppressing planktonic growth, suggesting potential use as an anti-biofilm adjuvant [73]. Table 2 compares phage-derived enzymes such as endolysins and depolymerases evaluated in food matrices, processing combinations, food-contact surfaces, or in vitro biofilm control models.

### 3.3. Indirect Effects on Bacterial Fitness

Beyond direct killing, phage exposure can indirectly reduce the fitness, persistence, or virulence of foodborne pathogens by selecting for bacterial variants with altered surface receptors or impaired resistance-associated traits [82]. Resistance to strictly lytic bacteriophages most often begins at the adsorption stage, where bacteria evade infection by modifying, masking, or losing the surface receptors required for phage attachment. Other mechanisms may include surface-glycan variation, extracellular barrier production, or intracellular defense systems that interfere with phage DNA entry, replication, or assembly [83]. However, resistance dynamics are strongly shaped by the surrounding environment. In a recent *Salmonella* study, reduced susceptibility arose mainly through receptor mutations in broth and cooked ham, whereas resistance in poultry was linked to the acquisition of large IncI1 plasmids encoding phage interference functions. Notably, resistance emerged in 92% of isolates after 24 h in broth, but only 4.3% of isolates from cooked ham stored at 4 °C lost susceptibility to at least two of three phages after 7 days, demonstrating that food matrices can substantially alter the frequency and mechanisms of resistance [84].

Phage resistance may also impose fitness costs that reduce bacterial virulence or persistence. In *L. monocytogenes* serovar 4b, phage-selected mutants defective in teichoic-acid galactosylation lost phage adsorption but also showed loss of surface-associated internalin B, impaired actin-tail formation, reduced host-cell invasion, and attenuated virulence in vivo [85]. Similar trade-offs have been reported in *S.* Enteritidis, where mutants resistant to a four-phage cocktail showed increased antibiotic susceptibility and reduced virulence compared with the wild-type strain [86]. Another study demonstrated that reduced phage sensitivity in *S.* Typhimurium was associated with markedly lower adsorption, decreased LPS content, and downregulation of virulence-related genes [87]. For *C. jejuni*, the reported fitness costs are variable. Some studies in broiler chickens found that phage-resistant isolates occurred at low frequency and showed reduced colonization, motility, and pathogenicity, whereas others detected resistant variants without reduced gut colonization capacity [88]. Similarly, *E. coli* biofilm survivors resistant to phage phT4A showed lower biofilm-forming capacity on plastic and stainless steel, suggesting that phage escape may reduce traits important for persistence on food-contact surfaces [70]. Collectively, these findings indicate that phage resistance should not be viewed only as a treatment failure. Resistance monitoring should assess the receptor involved, retained virulence, colonization ability, antimicrobial susceptibility, and biofilm-forming capacity. Rational cocktail design may therefore not only limit escape but also steer surviving populations toward less virulent, less persistent, or more treatable phenotypes [84].

## 4. Application of Bacteriophages in Food Safety

**Figure 4:**
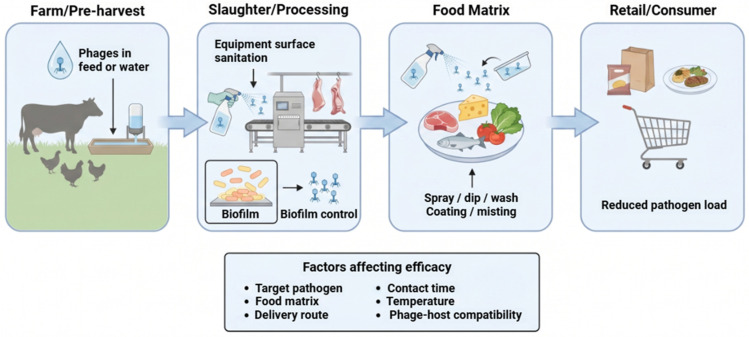
Stage-specific applications of bacteriophages in food safety. Bacteriophages may be applied at the pre-harvest, post-harvest, processing, and food-contact surface stages to reduce foodborne pathogens. Their efficacy depends on target pathogen, food matrix, delivery route, contact time, temperature, and phage–host compatibility (Created in https://BioRender.com, accessed on 9 May 2026).

Bacteriophage efficacy is strongly influenced by the point of contamination, target pathogen, food matrix, and delivery conditions. Therefore, phage-based interventions should be selected using a stage-specific framework rather than applied as a uniform control strategy. In pre-harvest settings, the main objective is to reduce pathogen carriage in live animals or associated farm reservoirs before slaughter or harvest. In contrast, post-harvest and processing applications focus on reducing contamination on food surfaces, in wash systems, on equipment, and in biofilms within processing environments [18,89]. Accordingly, identifying the primary contamination point is a critical first step in determining whether phages should be delivered via feed, water, gavage, spray, dip, mist, coating, or wash systems or through surface sanitation approaches [90]. This stage-specific structure is illustrated in Figure 4, which summarizes the major points at which bacteriophages can be incorporated across the food production continuum. In pre-harvest settings, phages may be administered through feed or drinking water to reduce pathogen carriage in food animals. During slaughter and processing, they can be applied to equipment and food-contact surfaces to support sanitation and biofilm control. At the food-matrix stage, phages may be delivered directly to foods through spraying, dipping, washing, coating, or misting approaches. Together, these interventions aim to reduce pathogen load before products reach retail outlets or consumers while emphasizing that efficacy is shaped by target pathogen, matrix type, delivery route, contact time, temperature, and phage–host compatibility.

### 4.1. Pre-Harvest Applications

Pre-harvest bacteriophage applications are designed to reduce pathogen carriage in food animals before slaughter, thereby lowering the microbial burden entering the processing chain. This strategy has been studied most extensively in commercial broiler production, particularly for *Campylobacter* and *Salmonella*, because intestinal colonization of poultry by these bacteria is a major source of carcass contamination during processing. For *Campylobacter*, experimental broiler studies have established proof of concept, with phage-treated birds showing reductions of approximately 0.5–5 log_10_ CFU/g in cecal contents, depending on the phage–host combination, dose, and timing of treatment [88]. Later work comparing oral gavage and in-feed administration found that both routes reduced fecal *Campylobacter* by approximately 2 log_10_ CFU/g, while in-feed delivery produced an earlier and more sustained response. However, phage-resistant phenotypes emerged at approximately 13%, emphasizing the importance of receptor compatibility and repeated-exposure ecology [91]. Field studies in commercial broiler houses further indicate that farm-level phage application is feasible, although reductions vary between barns and appear sensitive to timing, flock-specific strain composition, and the interval available for phage activity before slaughter [92].

For *Salmonella*, the poultry evidence is broader and increasingly relevant to production settings. Prophylactic administration of a six-phage cocktail through drinking water in newly hatched chicks reduced *S. enteritidis* cecal colonization by approximately 3 log_10_ during the early post-infection period without evidence of overt dysbiosis [93]. Feed-delivered cocktails have also reduced *Salmonella* colonization in challenged broilers, supporting feed as a scalable delivery route [94]. In addition to treating birds directly, phages may target farm-level reservoirs. For example, the UPWr_S134 cocktail reduced *S. enteritidis* biofilms on stainless steel and poultry drinker surfaces while largely preserving total viable surface communities, indicating targeted pathogen suppression without major disruption of the wider surface microbiota [68].

Pre-harvest applications in ruminants, particularly against STEC, remain more challenging. In experimentally inoculated sheep, oral administration of a cattle-derived phage cocktail reduced *E. coli* O157:H7 in feces within 24 h and lowered bacterial counts in intestinal sites, indicating potential for reducing shedding [95]. However, oral phage delivery in ruminants is complicated by gastric acidity, digestive enzymes, phage dilution, uneven transit, and the complexity of the gut ecosystem. Encapsulation and pH-responsive formulations may improve phage survival and intestinal delivery, especially for feed- or water-based administration [96,97]. These formulation challenges are particularly relevant for feed- or water-based use, because unprotected phages may lose infectivity under strongly acidic conditions before reaching the intended site of action [98].

Overall, pre-harvest phage use is limited by delivery route, dose, administration frequency, formulation stability, and integration with farm management practices. Gavage is useful for experimental purposes but poorly scalable, whereas feed and water delivery are more practical for commercial systems. Because in vivo phage replication is not guaranteed, dosing should consider host physiology, pathogen load, slaughter timing, and environmental re-exposure. Thus, pre-harvest phages are best positioned as targeted interventions during defined risk windows, complementing biosecurity, water and litter management, vaccination, and slaughter hygiene rather than replacing them [92,94]. Table 3 includes representative pre-harvest bacteriophage applications for reducing foodborne pathogen carriage in livestock and poultry.

### 4.2. Post-Harvest and Food Processing Application

Post-harvest applications represent the most advanced and translationally robust area of phage use in food safety, because phages can be applied directly to defined food surfaces, processing environments, or equipment at controlled titers and contact times. For *L. monocytogenes*, the bacteriophage P100 remains a well-characterized example. The application of 10^8^ PFU/g to raw salmon fillets produced pathogen reductions to 1.8, 2.5, and 3.5 log_10_ CFU/g, and also suppressed *Listeria* growth during refrigerated storage [57]. EFSA’s evaluation of Listex™ P100 similarly reported dose-dependent reductions of *L. monocytogenes* across ready-to-eat meat and poultry, fish and shellfish, and dairy products, with best-estimate mean reductions ranging from 1.7 to 3.4 log_10_ CFU at the highest tested dose [38]. Phage applications have also shown value against STEC on produce and meat. ECP-100 reduced *E. coli* O157:H7 on fresh-cut lettuce and cantaloupe during chilled storage, with treated samples showing substantially lower bacterial loads than controls [99]. Similarly, EcoShield™, a commercial three-phage preparation targeting *E. coli* O157:H7, significantly reduced contamination on beef and lettuce after a short contact time, although a single application did not protect foods against later recontamination [100]. These findings highlight both the practical utility and the limitations of post-harvest phages: they can reduce existing contamination but do not replace hygienic handling or prevent subsequent contamination events.

Phage efficacy remains strongly matrix dependent. In poultry, reductions of *Salmonella* and *Campylobacter* are often measurable but modest when phages are used alone, partly due to surface topography, organic residues, and limited phage–bacterium contact [38,99]. In seafood, selected *Vibrio parahaemolyticus* phages have shown stronger reductions under refrigerated conditions, suggesting particular value for cold-chain applications [101]. Therefore, phage efficacy should be validated in the intended food matrix rather than extrapolated from broth assays. Overall, post-harvest phages are best positioned as targeted decontamination tools within multi-hurdle food-safety systems. High local titer, uniform coverage, adequate contact time, and compatibility with refrigeration, organic acids, packaging, sanitation, or high-pressure processing are critical for reliable performance. Recent reviews on bacteriophage-loaded biopolymer films and active packaging further emphasize that incorporation or immobilization of phages into packaging materials may improve localized delivery, surface retention, and controlled contact with target bacteria. However, performance depends on polymer compatibility, phage stability, release kinetics, humidity, storage conditions, and the target food matrix [102,103]. Regulatory and safety assessments also emphasize that food-use phages should be strictly lytic, genomically characterized, free of toxin or AMR genes, and manufactured under controlled conditions [36]. Thus, the principal value of post-harvest phages lies in precise pathogen knockdown at defined control points, rather than broad-spectrum preservation or durable residual protection. Table 3 summarizes representative pre-harvest, post-harvest, and processing-environment applications, allowing comparison of reported efficacy and major limitations across livestock, poultry, produce, meat, seafood, ready-to-eat foods, and food-contact surfaces. Regulatory status and commercialization readiness were not listed separately for each example because these classifications are product-specific, region-dependent, and subject to change over time. Broader regulatory considerations, approved or commercially available phage products, and implementation barriers are discussed in Section 4.3.

**Table 3:** Representative pre-harvest and post-harvest bacteriophage applications for controlling foodborne pathogens across the food-production continuum.

Target Organism	Application Stage	Reported Efficacy	Main Limitations	References
*C. jejuni* in broiler chickens	Pre-harvest	Experimental broiler chicken studies reported reductions of approximately 0.5–5 log_10_ CFU/g in cecal contents, depending on phage–host combination, dose, and timing	Barn-to-barn variability; risk of resistance emergence; timing before slaughter is critical	[88]
in chicks	Pre-harvest	Prophylactic drinking water administration of a six-phage cocktail reduced cecal colonization by approximately 3 log_10_ during the early post-infection period, without overt dysbiosis	Most effective when started early; field durability and commercial-scale reproducibility require further validation	[93]
*Salmonella* on poultry drinkers/flock environment	Pre-harvest/environmental reservoir	UPWr_S134 reduced *S. enteritidis* on poultry drinker surfaces; treated drinkers had no detectable *S. enteritidis* by day 9, while total viable counts were broadly maintained	Surface-focused rather than systemic control; requires integration with cleaning, water hygiene, and biosecurity	[68]
*E. coli* O157:H7 (STEC) in sheep and cattle models	Pre-harvest	Sheep studies showed approximately 100-fold reduction in gastrointestinal *E. coli* O157:H7; feedlot-cattle studies indicate that oral/rectal phage delivery can affect shedding dynamics	Ruminant gut conditions, acid exposure, uneven transit, and ecological complexity reduce predictability	[104,105]
*Salmonella* spp. in pigs/swine	Pre-harvest	Oral or feed-delivered phage cocktails have been evaluated as short-term interventions to reduce *Salmonella* colonization or infection in pigs before slaughter	Limited in vivo and field-scale validation; efficacy depends on delivery route, phage stability during gut passage, timing before slaughter, strain-specific host range, and re-exposure during transport or lairage	[106,107]
*L. monocytogenes* on raw salmon	Post-harvest	LISTEX^TM^ P100 at 10^8^ PFU/g produced 1.8, 2.5, and 3.5 log_10_ CFU/g reductions depending on initial inoculum and also suppressed growth during refrigerated storage.	Strong matrix and inoculum effects; high local dose and uniform surface coverage are required	[57]
*L. monocytogenes* on ready-to-eat (RTE) foods	Post-harvest/processing	EFSA reported dose-dependent reductions in RTE meat/poultry, fish/shellfish, and dairy products, with estimated mean reductions of 1.7–3.4 log_10_ CFU at the highest tested dose	Efficacy is product- and plant-specific; monitoring for P100 susceptibility is recommended	[38,108]
*E. coli* O157:H7 (STEC) on produce and beef	Post-harvest	ECP-100 reduced *E. coli* O157:H7 on fresh-cut lettuce and cantaloupe during chilled storage; EcoShield^TM^ reduced contamination on beef by ≥94% and lettuce by 87% after 5 min	Limited residual protection; one-time application does not protect against later recontamination.	[99,100]
*Salmonella* on chicken meat	Post-harvest	Reported reductions are often measurable but modest when phages are used alone; some poultry studies reported sub-log to low-log reductions depending on dose, storage, and surface conditions	Poultry skin/meat topography, organic matter, moisture distribution, and limited phage–bacterium contact reduce efficacy	
*C. jejuni* on chicken skin	Post-harvest	Host-specific phages reduced recoverable *C. jejuni* on experimentally contaminated chicken skin; reductions were influenced by phage dose, storage temperature, and surface conditions	Cooling, moisture, skin topography, and attachment efficiency affect recovery and performance	[109]
*Vibrio parahaemolyticus* on raw fish/salmon	Post-harvest	VPT02-type phages have reported strong reductions on raw fish slices; cold-adapted phage XY75 reduced *V. parahaemolyticus* in salmon by >5.98 log_10_ CFU/g within 6 h at 4 °C	Often requires high multiplicity of infection (MOI), strong cold-chain control, and validation across seafood matrices	[63]
*E. coli* O157:H7 (STEC) on hard surfaces and produce	Post-harvest/ Processing environment	ECP-100 reduced *E. coli* O157:H7 on hard surfaces, tomato, spinach, broccoli, and ground beef, with reductions depending on phage concentration and surface type	Surface type, organic matter, moisture, and recontamination risk affect durability	[110]

* Note: “No detectable” indicates that the organism was below the detection limit of the method used in the cited study and does not necessarily indicate complete eradication. Abbreviations: CFU, colony-forming units; CFU/g, colony-forming units per gram; EFSA, European Food Safety Authority; PFU, plaque-forming units; PFU/g, plaque-forming units per gram; RTE, ready-to-eat; STEC, Shiga toxin-producing *Escherichia coli*.

### 4.3. Commercial Phage Products and Regulatory Status

The United States has one of the most developed regulatory frameworks for bacteriophage applications in food safety. The FDA permits the use of a *L. monocytogenes*-specific bacteriophage preparation as a direct food additive for ready-to-eat meat and poultry products under 21 CFR §172.785, where the additive is defined as a mixture of six purified, lytic-type bacteriophages lacking lysogenic activity [36]. Beyond this food-additive rule, several FDA GRAS notices and food-contact notifications cover phage preparations targeting *Listeria, Salmonella*, STEC, and *Campylobacter*. Examples include GRN 198 for phage P100 on cheeses, GRN 528 for *Listeria*-specific phages on fish, shellfish, fruits, vegetables, and dairy products, FCN 1018 for EcoShield use on red-meat parts and trim before grinding, GRN 435 and GRN 468 for *Salmonella*-specific phages across meat, poultry, seafood, produce, and grain products, GRN 724 for STEC-specific phages on beef carcasses, and GRN 966 for *C. jejuni*-specific phages on raw red meat and poultry [111]. In parallel, the USDA Food Safety and Inspection Service (FSIS) lists selected bacteriophage preparations as safe and suitable for specified meat and poultry applications, including *Salmonella*-targeted preparations used on poultry and red meat products [112].

In the (EU, regulatory development has been more gradual and product-specific. EFSA’s 2009 BIOHAZ assessment concluded that, under defined conditions, some bacteriophages may be effective for targeted pathogen reduction in meat, milk, and related products, but also emphasized uncertainty regarding protection against recontamination [108]. In 2016, EFSA issued a positive safety and efficacy assessment for Listex™ P100 for reducing *L. monocytogenes* in ready-to-eat meat and poultry, fish and shellfish, and dairy products [38]. More recently, Commission Implementing Regulation (EU) 2025/1390 authorized the preparation of the bacteriophages PCM F/00069, PCM F/00070, PCM F/00071, and PCM F/00097 as a zootechnical feed additive for poultry, establishing EU-level authorization for BAFASAL^®^ use in complementary feed and drinking water [113]. The European Medicines Agency (EMA) guidance addresses phages in a different regulatory context, where veterinary medicinal products designed for phage therapy are under Regulation (EU) 2019/6, rather than food-processing or feed-additive applications [114].

Regulatory pathways outside the United States and the EU also differ by jurisdiction. Australia and New Zealand have evaluated phage preparations as processing aids through FSANZ, whereas Canada relies on a case-by-case processing-aid framework in which Health Canada may issue “no objection” opinions for antimicrobial processing aids rather than using a United States-style GRAS system [115]. Commercially, widely cited food-safety phage products include Listex™/PhageGuard L/ListShield for *Listeria* [116], EcoShield- or ShigaShield-type preparations for pathogenic *E. coli* [117], SalmoFresh/Salmonelex/PhageGuard S for *Salmonella* [118], CampyShield-type preparations for *Campylobacter* [119], and BAFASAL^®^ for poultry-associated *Salmonella* [120]. However, brand names alone are insufficient to determine permitted use. The controlling factors are the jurisdiction, regulatory clearance, target pathogen, intended matrix, application method, and label claims.

## 5. Phage Resistance in Foodborne Pathogens

### 5.1. Mechanisms of Bacterial Resistance to Phages

#### 5.1.1. Receptor Modification, Masking, or Loss

Receptor modification, masking, or loss remains the most important phage-resistance mechanism in foodborne bacteria, as it blocks infection at the adsorption stage. In *C. jejuni*, phase-variable changes in capsular polysaccharide structures, particularly O-methyl phosphoramidate (MeOPN) modification, can alter phage receptor availability and prevent adsorption by Fletchervirus-type phages. Comparative analysis of Danish broiler isolates of *C. jejuni* showed that strains belonging to the clonal complex (CC) ST-21 encoded a phase-variable MeOPN receptor and are susceptible to Fletchervirus phages, whereas ST-45 strains lack this receptor and exhibit a different susceptibility pattern [121]. In *S. enterica*, phase-variable expression of *opvAB* operon alters the length of LPS O-antigen chain and confers resistance to O-antigen-targeting phages such as P22, 9NA, and Det7, although this resistance is associated with reduced virulence and is reversible when phage selection is removed [122]. In *L. monocytogenes*, resistance is frequently mediated by altered cell wall teichoic acid glycosylation due to a mutation in *gtcA*. This gene is required for serotype-specific teichoic-acid glycosylation in serotype 4b strains and its disruption prevents adsorption by both genus-specific and serotype 4b-specific phages [123].

#### 5.1.2. Restriction-Modification Systems

Restriction–modification systems act after phage DNA has entered the bacterial cell. In these systems, restriction endonucleases cleave foreign phage DNA lacking the appropriate host-specific methylation pattern, whereas cognate methyltransferases protect bacterial self-DNA. A foodborne pathogen-relevant example has been described in epidemic clone II *L. monocytogenes*, where low-temperature phage resistance was linked to a type II restriction–modification system encoded by open reading frames (ORFs) with homology to restriction endonuclease and methyltransferase genes; deletion of the restriction-associated gene restored phage susceptibility at both 25 °C and 37 °C [124]. A second example comes from broiler chicken-derived *C. jejuni*, where deletion of *mcrB*, encoding a methyl-specific McrBC endonuclease component, rendered a previously resistant strain susceptible to multiple Fletchervirus and Firehammervirus phages, indicating that methylation-dependent DNA restriction can contribute substantially to phage resistance in poultry-associated *Campylobacter* [121].

#### 5.1.3. CRISPR–Cas-Mediated Adaptive Immunity

CRISPR-Cas systems provide adaptive, sequence-specific antiphage immunity through acquisition of phage-derived spacers, transcription of CRISPR RNAs, and guide-directed cleavage of matching invasive nucleic acids. In *Campylobacter*, DA10-like temperate phages are strongly represented in type II-C CRISPR arrays. One study found that approximately 75% of DA10 open reading frames were represented as ~30-bp spacers in diverse *Campylobacter* CRISPR arrays, suggesting long-term CRISPR-mediated pressure against these phages [125]. However, CRISPR-Cas does not account for all resistances to contemporary lytic *Campylobacter* phages, because broiler isolates with distinct susceptibility profiles may carry CRISPR spacers that do not match the tested phages, while other barriers such as MeOPN receptor variation and McrBC-like restriction systems, also contribute to resistance [121].

#### 5.1.4. Abortive Infection Systems

Abortive infection acts at later stages of the phage life cycle by preventing completion of phage replication after infection has already begun. In this mechanism, infected cells undergo growth arrest, metabolic shutdown, or death before phage maturation and release, thereby sacrificing individual cells to protect the surrounding bacterial population. Direct functional evidence is strongest in enterobacterial models. For example, toxin–antitoxin–chaperone systems such as HigBAC and CmdTAC in *E. coli* can sense phage infection and restrict phage propagation, with HigBAC triggered by the phage λ major tail protein and CmdTAC inhibiting translation through toxin-mediated modification of mRNA [126]. Although these systems have been characterized mainly in model enterobacteria, the principle is relevant to foodborne Enterobacteriaceae because abortive infection can convert single-cell sacrifice into population-level protection and reduce the amplification of lytic phages in food, animal, or processing-environment contexts. The major bacterial defense mechanisms that reduce phage susceptibility in foodborne pathogens and the typical genetic and phenotypic changes and implications for control are summarized in Table 4.

### 5.2. Consequences of Phage Resistance

The consequences of phage resistance are context-dependent. While resistance may reduce treatment efficacy, it can also impose fitness costs, because phage receptors often contribute to nutrient uptake, membrane integrity, motility, biofilm formation, immune evasion, or host-cell adhesion. Thus, receptor changes that prevent phage adsorption may simultaneously reduce bacterial competitiveness, virulence, persistence, or AMR [28,86]. In *L. monocytogenes*, resistance linked to the loss of cell wall teichoic acid glycosylation can prevent phage adsorption but also disrupt surface-associated virulence factors such as internalin B, leading to impaired host-cell invasion and attenuated virulence [85]. Similarly, in *S.* Enteritidis, resistance to a receptor-diverse phage cocktail delayed resistance emergence and selected mutants with increased antibiotic susceptibility and reduced virulence. These findings suggest that rational cocktail design can sometimes steer bacterial evolution toward less fit or more treatable phenotypes [86]. However, resistance outcomes vary by environment. In *Salmonella*, resistance frequency and mechanisms differed between broth, cooked ham, and broiler chickens, indicating that laboratory models may overestimate resistance compared with refrigerated food matrices [84]. Therefore, phage resistance should be considered a predictable but manageable feature of phage biocontrol. Monitoring should include receptor changes, cross-resistance, growth and stress tolerance, biofilm formation, antibiotic susceptibility, and virulence traits. Sustainable application will require receptor-diverse cocktails, phage rotation, combination with other hurdles, and surveillance of resistant phenotypes.

## 6. Strategies to Overcome Phage Resistance

### 6.1. Phage Cocktails

Phage cocktails are a central strategy for reducing resistance because simultaneous escape from multiple phages is less likely when the component phages recognize distinct bacterial receptors. Accordingly, recent work has shifted from empirical phage mixing toward rational cocktail design based on host range, genetic diversity, receptor usage, and phage resistance profiles. In *S.* Enteritidis, a four-phage cocktail targeting LPS O-antigen, LPS outer and inner core structures, and the outer-membrane proteins BtuB and TolC delayed resistance emergence more effectively than single phages. Importantly, cocktail-resistant mutants showed increased antibiotic susceptibility and reduced virulence [86]. Similarly, a multireceptor phage cocktail reduced *Salmonella* loads on chicken skin by 3.5 log_10_ CFU/cm^2^ at 15–25 °C and 2.5 log_10_ CFU/cm^2^ at 4 °C, supporting receptor diversity as a practical design principle for food biocontrol [127].

Food-matrix studies further support cocktails as the preferred post-harvest approach. A broad-spectrum three-phage cocktail reduced nontyphoidal *Salmonella* on raw chicken breast by >3.2 log_10_ after 5 days at 10 °C and >1.7 log_10_ after 16 h at 22 °C, demonstrating applicability under poultry-relevant storage conditions [128]. However, a broad host range alone does not guarantee resistance control; cocktail components should ideally target different receptors and be evaluated individually for lytic activity, genome safety, stability, and transduction potential. In the multireceptor *Salmonella* phage cocktail study, one tested phage showed low-frequency transduction comparable to phage P22, highlighting the need for component-level safety assessment rather than evaluation of the final mixture alone [127]. Thus, for food applications, phage cocktails should be designed as receptor-diverse, genomically safe, and matrix-validated interventions rather than simple combinations of broadly active phages. Several phage cocktails have demonstrated pathogen reduction across poultry meat, ready-to-eat foods, produce, and seafood matrices; however, their effectiveness varies according to receptor diversity, phage dose, matrix structure, temperature, and contact time (Table 5).

### 6.2. Phage–Antibiotic Synergy (PAS)

Phage–antibiotic synergy (PAS) describes combinations in which bacteriophages and antibiotics produce greater antibacterial activity together than either agent alone. However, PAS is not a universal phenomenon; it depends on the specific phage, bacterial host, antibiotic mechanism of action, antibiotic concentration, and phage-to-bacterium ratio. Mechanistic work has shown that sublethal antibiotic exposure can induce bacterial filamentation, delay phage-mediated lysis, and increase intracellular phage production, thereby enhancing plaque size and antibacterial activity [133]. Broader interaction-mapping studies further showed that PAS is governed by the antibiotic mechanism of action and treatment stoichiometry. Liu et al. demonstrated that some phage–antibiotic pairings are synergistic, whereas others are additive or antagonistic. Under selected conditions, phages also lowered the effective minimal inhibitory concentration (MIC) against drug-resistant *E. coli*. Therefore, PAS should be empirically mapped for each phage–host–drug combination rather than inferred from antibiotic class alone [134].

In foodborne pathogens, direct PAS evidence is promising but remains less mature than phage-cocktail evidence. Recent studies with multidrug-resistant (MDR) *S.* Typhimurium suggest that combining phage cocktails with ciprofloxacin can improve bacterial killing, delay resistance emergence, enhance biofilm eradication, and increase antibiotic sensitivity; however, these data are mainly derived from infection-model contexts rather than food-matrix applications [135]. Thus, PAS currently has stronger relevance for pre-harvest, veterinary, or therapeutic settings than for routine post-harvest food processing. Overall, PAS and receptor-diverse cocktail design should be viewed as complementary resistance-management strategies. PAS may enhance killing or reduce the effective antibiotic burden in selected contexts, whereas receptor-aware cocktails may channel bacterial escape toward antibiotic-sensitive or attenuated phenotypes [86,134]. Nevertheless, because PAS outcomes can range from synergy to antagonism, application in foodborne pathogen control requires systematic validation under realistic biological or food-chain conditions.

### 6.3. Engineered and Synthetic Phages

Engineered phages are designed to overcome bacterial resistance through two main approaches: expanding or restoring host adsorption and introducing additional intracellular killing mechanisms. Host-range engineering has already shown strong proof-of-concept value. In the T3 “phagebody” platform, structure-guided mutagenesis of tail-fiber host-range regions generated diverse phage variants with altered bacterial specificity. Importantly, the selected variants maintained antibacterial activity over extended periods, with no detectable emergence of resistance, and remained effective in a mouse wound model [136]. Similarly, in *Salmonella*, engineering of *Ackermannviridae* tailspike proteins in the reporter phage SPTD1.NL produced the RBP-SPTD1-3 construct, which broadened detection across additional *Salmonella* serovars while reducing off-target recognition of *Citrobacter* [137]. Together, these studies demonstrate that receptor engineering can provide precise and functionally meaningful host-range expansion, particularly when natural phage activity is too narrow or when resistance can arise through simple receptor-based escape.

The most advanced engineered-phage platforms combine receptor engineering with CRISPR-based antibacterial functions. In the SNIPR001 program, researchers screened 162 wild-type *E. coli* phages and selected phages with complementary host ranges and receptor targets. These phages were then further modified using tail-fiber engineering and type I-E CRISPR–Cas arming. For example, tail-fiber modification of the CRISPR-armed phage α15.2 changed it from an LPS-dependent phage into a dual-receptor phage that could infect *E. coli* through both LPS and Tsx, an outer-membrane nucleoside transporter that can also serve as a phage receptor. The CRISPR module provided targeted antibacterial activity, and the final four-phage CRISPR-armed cocktail performed better than the individual phages in mouse gut models, reduced the emergence of phage-tolerant bacteria, and entered clinical development [138]. Similarly, P1-derived CRISPR–Cas9 phagemids achieved sequence-specific killing of *Shigella flexneri* and reduced bacterial burden in zebrafish larvae [139]. More recently, an engineered *S.* Typhimurium phage displaying LL-37 on the virion surface reportedly prevented resistance development, reduced adhesion and invasion, and improved *Galleria mellonella* survival [140]. Collectively, these studies show that phage engineering can address resistance at multiple levels, including receptor escape, intracellular DNA destruction, and collateral anti-virulence effects.

For food applications, the main barrier for engineered phages is no longer proof of concept, but regulatory and manufacturing feasibility. The current United States food-use regulation remains centered on natural, strictly lytic phages. For example, the *Listeria*-specific food additive under 21 CFR §172.785 is defined as six individually purified lytic-type phages with identity, titer, lytic activity, and toxin-related specifications [36]. In contrast, engineered phages would likely require additional review under biotechnology or genetically modified organisms (GMOs) related frameworks. EFSA places genetically modified microorganisms intended for food or feed within a dedicated risk-assessment pathway, while United States biotechnology oversight is coordinated across the FDA, EPA, and USDA depending on the product and its intended use [141,142]. Although EMA guidance indicates that genetically or chemically modified phages, including synthetic-genome phages, are being considered within medicinal-product frameworks, this pathway is primarily relevant to therapeutic rather than food-processing applications. Therefore, engineered phages currently represent a scientifically powerful anti-resistance strategy, but they remain less immediately deployable in food systems than natural lytic phage cocktails. Representative engineered phage platforms, including tail-fiber modification, chimeric tailspike engineering, CRISPR–Cas-armed phages, phagemid delivery systems, and antimicrobial peptide-displaying phages, are summarized in Table 6.

Taken together, resistance-mitigation strategies differ substantially in their practical readiness for food-production settings. Receptor-diverse natural phage cocktails, phage rotation, resistance surveillance, and integration with established hurdles such as refrigeration, sanitation, organic acids, packaging, or high-pressure processing are currently the most directly translatable approaches because they can be incorporated into existing food-safety systems with relatively limited regulatory disruption. In contrast, engineered phages, CRISPR–Cas-armed phages, and phagemid-based platforms offer powerful tools for expanding host range or suppressing resistance, but their food-chain applications remain less immediate because of unresolved questions related to genetic stability, environmental release, regulatory classification, consumer acceptance, and large-scale manufacturing. Therefore, resistance management should not rely on a single strategy; practical implementation should combine receptor-informed phage selection, matrix-specific validation, post-application surveillance, and integration with Hazard Analysis Critical Control Point (HACCP)-based or multi-hurdle control programs.

## 7. Challenges and Limitations of Phage-Based Biocontrol

Despite increasing regulatory and commercial interest, phage-based biocontrol remains a context-dependent intervention rather than a universally predictable food-safety technology. Its efficacy is strongly influenced by host range, pathogen strain diversity, food matrix composition, temperature, moisture availability, surface structure, and application conditions. The EFSA concluded that bacteriophages can be effective under specific conditions, but their efficacy depends on the food, phage type, method of use, and environmental factors, and the available evidence does not support reliable protection against recontamination after treatment [108,143]. This variability indicates that phage efficacy should be interpreted as matrix- and application-specific rather than universally transferable across pathogens, products, or processing environments.

A major biological limitation is the narrow and often strain-specific host range of phages, which can limit portability across processing plants, poultry and livestock operations, and outbreak strains. Phage resistance may also emerge through receptor loss or modification, altered surface polysaccharides, restriction–modification systems, CRISPR-Cas immunity, abortive infection, or other intracellular defense systems. Importantly, the resistance risk is not constant across environments. Therefore, resistance monitoring should be incorporated into phage validation and post-application surveillance, particularly when phages are used repeatedly in farms, processing plants, or biofilm-prone environments. Food matrices can further limit phage performance by reducing their diffusion, adsorption, and access to bacterial cells. Low water activity, high fat or protein content, surface crevices, organic residues, and refrigeration can restrict phage movement or reduce bacterial metabolic activity, thereby limiting productive infection [144]. Biofilms are especially difficult targets for phages. A systematic review and meta-analysis of foodborne pathogen biofilms on stainless steel found an overall mean reduction of 38% after phage treatment, but older biofilms and low-temperature conditions were significantly less responsive [145].

Technical and manufacturing challenges also affect industrial translation. A successful application requires a sufficiently high local titer, uniform surface coverage, adequate contact time, and stability during storage, processing, and distribution. Formulation remains important, especially for feed- or water-based delivery, refrigerated foods, dry surfaces, and complex matrices where phages may lose activity due to acidity, enzymes, low moisture, organic matter, or limited diffusion. In addition, commercial preparations require robust production, purification, potency testing, genomic characterization, and control of host-cell residues or contaminants. These expectations are reflected in the United States regulations for *Listeria*-specific phage preparations, which require purified lytic-type phages lacking lysogenic activity, defined phage titers, lytic activity, absence of toxin-encoding sequences, and microbiological purity [36].

Regulatory classification remains another important limitation. In the United States, phage products may be regulated through food additive, GRAS, food-contact, or USDA-FSIS safe-and-suitable pathways, depending on their intended use [141]. In the EU, EFSA has provided scientific opinions on the use of phages in food, but classification and authorization remain more product- and use-specific [143]. ANSES, the French Agency for Food, Environmental and Occupational Health & Safety, also emphasized that phages should be considered supplementary control tools rather than replacements for good hygiene practices or HACCP-based systems [146]. Engineered or synthetic phages are likely to face additional regulatory scrutiny because EMA guidance for veterinary phage products requires addressing quality, safety, efficacy, target-animal safety, user safety, and environmental risk considerations [114]. In addition, large-scale implementation remains limited by the need for multi-site validation under realistic commercial conditions, including naturally contaminated samples, repeated applications, plant-to-plant variability, cost-effectiveness, and compatibility with existing sanitation and HACCP-based systems [147]. Overall, the main limitations of phage biocontrol include narrow host ranges, resistance emergence, matrix-dependent efficacy, incomplete biofilm eradication, formulation and stability constraints, manufacturing quality control, regulatory fragmentation, and limited large-scale field validation. Therefore, future implementation should prioritize local host-range testing, multi-receptor phage cocktails, matrix-specific validation, standardized efficacy endpoints, genomic and transduction safety screening, scalable manufacturing, large-scale field trials, and post-application monitoring for resistance or efficacy drift under commercial food-production conditions.

## 8. Future Perspectives and Research Gaps

Future research should prioritize standardized, matrix-specific validation of phage biocontrol under realistic food-processing conditions, including relevant pathogen loads, storage temperatures, contact times, and recontamination scenarios. Key remaining research gaps include limited multi-site commercial validation, incomplete understanding of long-term resistance dynamics, insufficient matrix-specific stability data, and the absence of balanced regulatory expectations for rapidly updated phage cocktails or engineered phage platforms. Because phage efficacy varies by pathogen, food matrix, delivery method, and environmental conditions, results from broth or simplified laboratory models should not be directly extrapolated to commercial foods. Rational phage-cocktail design based on local strain panels, receptor diversity, genomic safety, and resistance monitoring will be essential to broaden host range and limit bacterial escape. Additional work is needed to improve formulation stability, scalable manufacturing, quality control, and compatibility with existing hurdles such as refrigeration, sanitation, packaging, organic acids, or high-pressure processing. Engineered and synthetic phages may offer future advantages, but their food-chain application will require clearer regulatory pathways and careful assessment of genetic stability, environmental safety, and consumer acceptance. Broader use of phages, particularly engineered phages, should also be accompanied by ecological surveillance to evaluate effects on microbial community structure, non-target bacterial populations, phage persistence, horizontal gene transfer risk, and long-term resistance dynamics. Such monitoring is especially important in farms, processing environments, wastewater, manure, and other food-chain-associated reservoirs where repeated phage exposure could influence bacterial population structure and evolutionary trajectories. Overall, future progress will depend on harmonized efficacy standards, large-scale field trials, post-application resistance surveillance, and integration of phages into validated multi-hurdle food-safety systems rather than their use as stand-alone interventions. Future research should also integrate phage engineering, AI-assisted phage selection, real-time resistance monitoring, scalable and quality-controlled manufacturing, economic feasibility assessment, and internationally harmonized regulatory standards to support broader translation of phage-based food-safety interventions.

## 9. Conclusions

Bacteriophages represent a promising, targeted approach for improving food safety, particularly against major bacterial pathogens such as *Salmonella*, *Campylobacter*, STEC, *L. monocytogenes*, and *V. parahaemolyticus*. Their specificity, ability to disrupt biofilms, compatibility with multi-hurdle strategies, and expanding regulatory acceptance support their use across pre-harvest, post-harvest, and processing-environment applications. However, phage efficacy remains highly context-dependent and is influenced by host range, food matrix, delivery route, dose, contact time, environmental conditions, and bacterial resistance. Therefore, successful implementation will require carefully selected strictly lytic phages, rational cocktail design, genomic safety screening, matrix-specific validation, scalable manufacturing, and continued surveillance for resistance or efficacy drift. Overall, bacteriophages should be viewed not as replacements for existing food-safety systems but as precision tools that can strengthen integrated One Health approaches to controlling foodborne pathogens.

## Figures and Tables

**Table 1:** Representative food biocontrol bacteriophages with reported replication kinetics and food-matrix efficacy.

Phage Designation	Target Pathogen	Genome Class/Characteristics	Latent Period	Burst Size	Reported Reduction in Specific Food Matrices	References
P100/LISTEX™ P100	*L. monocytogenes*	Strictly lytic tailed dsDNA phage; classified as *Caudovirales*, *Myoviridae*/SPO1-like in regulatory assessments	NR ^¥^	NR ^¥^	Raw salmon fillet: approximately 1.8–3.5 log_10_ CFU/g reduction depending on inoculum/load and treatment conditions; soft cheese: approximately 2.1–2.3 log_10_ reduction within 30 min	[39,57,62]
vB_LmoP_M15	*L. monocytogenes*	Lytic tailed dsDNA phage; genome approximately 48.5 kb	15 min/15–20 min	172 PFU/cell	Pasteurized milk: treated samples reached approximately 5.1 log_10_ CFU/mL compared with 7.55 log_10_ CFU/mL in untreated controls by day 7, representing about 2.45-log_10_ attenuation	[56]
L223	*S.* Typhimurium/*Salmonella* spp.	Lytic dsDNA *Salmonella* phage	30 min	515 PFU/cell	Chicken breast: 2.17 log_10_ CFU/piece reduction at 4 h under high-dose treatment	[59]
SQ17/vB_EcoM_SQ17	EHEC O157:H7 and ETEC	Lytic dsDNA myophage; no toxin, virulence, lysogeny, or AMR genes reported	10 min	71 PFU/cell	EHEC O157:H7 on lettuce: 2.23–3.83 log_10_ CFU/piece reduction; raw beef: up to 2.35 log_10_ reduction; milk at 4 °C: reduced to below detection limit	[60]
vB_EcoM-ECP26	EHEC/STEC O157:H7	Lytic dsDNA myovirus	55 min	1914 PFU/cell	Romaine lettuce at 4 °C: 0.9 log immediate reduction, 1.2 log_10_ reduction by day 3, and undetectable level by day 5	[61]
VPT02	*V. parahaemolyticus*	Lytic dsDNA phage; genome 120,547 bp; no toxin or AMR genes reported	20 min	208 PFU/cell	Ready-to-eat raw fish flesh slices: up to 3.9 log_10_ reduction compared with untreated control	[58]
XY75	*V. parahaemolyticus*	Cold-adapted lytic dsDNA phage; no virulence or AMR genes reported	5 min	118 PFU/cell	Salmon at 4 °C: >5.98 log_10_ CFU/g reduction within 6 h	[63]

Note: NR ^¥^—not reported in the food-application source. Abbreviations: AMR, antimicrobial resistance; CFU, colony-forming units; dsDNA, double-stranded DNA; EHEC, enterohemorrhagic *Escherichia coli*; ETEC, enterotoxigenic *Escherichia coli*; kb, kilobase; bp, base pairs; PFU, plaque-forming units; STEC, Shiga toxin-producing *Escherichia coli*. L., S., and V. denote the genera *Listeria*, *Salmonella*, and *Vibrio*, respectively.

**Table 2:** Phage-derived enzymes evaluated for food safety, biofilm control, and food-contact surface applications.

Enzyme	Source Phage	Enzyme Type/Principal Substrate Specificity	Demonstrated Effect	Application Status *	References
PlyP100	*Listeria* phage P100	Endolysin; amidase activity against directly cross-linked Gram-positive peptidoglycan, especially *Listeria* spp.	In Queso Fresco, PlyP100 showed antilisterial activity during refrigerated storage; When combined with nisin, *L. monocytogenes* reached non-enumerable levels after 4 weeks, with no resistance detected to PlyP100 or nisin	Food-matrix proof-of-concept	[74]
PlyP40/PlyPSA	*Listeria* phages P40 and PSA	Endolysins targeting *Listeria* peptidoglycan	Reduced *L. monocytogenes* counts in Queso Fresco; PlyP40 lowered counts over the 28-day shelf-life, while PlyPSA lowered counts until day 14; neither outperformed PlyP100	Food-matrix proof-of-concept	[75]
Ply511/PlyP40/PlyP825	*Listeria* phages 511, P40, and P825	Endolysins targeting *Listeria* peptidoglycan	In buffer, combining endolysins with high hydrostatic pressure produced strong synergistic killing; for example, PlyP825 plus 300 MPa for 1 min reduced *L. monocytogenes* by 5.5 log_10_ CFU, whereas each treatment alone caused only minor reductions	Process-combination proof-of-concept, initially buffer-based	[76]
PlyP825	*Listeria* phage P825	Endolysin targeting *Listeria* peptidoglycan	In food models, including milk and mozzarella, PlyP825 combined with high hydrostatic pressure improved inactivation of *L. monocytogenes* and supported milder pressure processing; effects were food-matrix dependent and were not equally effective in smoked salmon	Food-process combination proof-of-concept	[77]
Dpo10	*E. coli* O157:H7 siphophage BECP10	Depolymerase/tailspike protein targeting O157 O-polysaccharide/LPS; predicted pectate lyase activity	Did not directly inhibit planktonic growth but degraded O-polysaccharide, increased serum sensitivity, inhibited biofilm formation 8-fold on polystyrene, and reduced biofilm formation by 2.56 log_10_ CFU/coupon on stainless steel	Food-contact surface proof-of-concept	[73]
P22 tailspike protein	*Salmonella* phage P22	Tailspike endorhamnosidase recognizing and cleaving *Salmonella* O-antigen repeats in LPS	Serves as a mechanistic benchmark for receptor binding and receptor destruction; P22 tailspike recognizes *Salmonella* O-antigen repeats; endorhamnosidase activity linked to receptor degradation and DNA ejection biology	Mechanistic benchmark; not a direct food-process product	[78,79,80]
Dpo52	*S. enteritidis phage* vB_Sen_S_3_P	Depolymerase encoded by ORF52; extracellular polysaccharide-degrading activity against biofilm-associated surface polysaccharides	Inhibited biofilm formation of carbapenem-resistant *S. enteritidis* through extracellular polysaccharide degradation; Dpo52 was stable across pH 4–11 and 4–60 °C and was non-cytotoxic to macrophages in the tested model	Early in vitro/pre-application stage	[81]

* Note: Application status was classified based on the level of translational evidence. “Proof-of-concept” indicates experimental validation in food matrices, food-contact surfaces, or processing-combination models, but not routine commercial deployment. “Early in vitro/pre-application stage” indicates activity demonstrated mainly in buffer systems, laboratory assays, or model biofilms. “Mechanistic benchmark; not a direct food-process product” indicates an enzyme or phage-derived component used primarily to illustrate receptor binding, substrate specificity, or mechanistic principles, rather than a technology currently intended for direct food-processing application. “Near-commercial” or “commercially deployed” should be used only when regulatory authorization, product availability, or validated industrial-scale use has been demonstrated. Abbreviations: CFU, colony-forming units; Dpo, depolymerase; LPS, lipopolysaccharide; MPa, megapascal; ORF, open reading frame; Ply, phage lysin/endolysin.

**Table 4:** Major mechanisms of phage resistance in foodborne bacteria and their implications for phage-based biocontrol.

Bacterial Defense Mechanism	Typical Genetic/Phenotypic Change	Implications for Control	References
Receptor modification or loss	Mutation, phase variation, masking, or loss of LPS O-antigen, capsular polysaccharide (CPS), cell wall teichoic acid (WTA), flagella, pili, porins, or efflux-associated receptors	Most common resistance route; may generate cross-resistance if phages share the same receptor; mitigate using cocktails targeting distinct receptors and combining phages with non-phage hurdles	[28,85,123]
Restriction–modification systems	Acquisition, activation, or temperature-dependent expression of restriction endonuclease–methyltransferase modules	Sequence-specific intracellular barrier; lineage- and temperature-dependent effects may cause matrix-specific failure; mitigate by local strain testing, adapted phages, and receptor-diverse cocktails	[28,121,124]
CRISPR–Cas systems	Spacer acquisition, protospacer/ protospacer adjacent motif (PAM) recognition, and Cas-mediated cleavage of phage nucleic acids	Highly sequence-specific; phage mutation, protospacer loss, PAM alteration, or anti-CRISPR activity may erode efficacy; useful for genomic surveillance of likely resistance routes	[28,121]
Abortive infection systems	Activation of toxin–antitoxin, toxin–antitoxin–chaperone, or related suicide/dormancy modules after phage infection	Reduces phage replication and burst size rather than adsorption; may coexist with receptor or DNA-defense mechanisms; difficult to predict phenotypically, so cocktails and hurdle integration remain important	[28]

* Note: “Receptor-diverse cocktails” refers to phage combinations that target different bacterial surface receptors to reduce the likelihood of cross-resistance; “Hurdle integration” refers to combining phages with additional antimicrobial or processing interventions, such as organic acids, bacteriocins, high pressure, refrigeration, sanitation, or other food-safety controls. Abbreviations: CRISPR–Cas, clustered regularly interspaced short palindromic repeats–CRISPR-associated protein system; LPS, lipopolysaccharide.

**Table 5:** Representative phage cocktails evaluated for controlling foodborne pathogens in food applications.

Cocktail Name/Product	Target	Component Phages or Receptor Targets	Reported CFU/log Reductions in Food Matrices	References
ListShield™	*L. monocytogenes*	6 lytic phages: LIST-36, LMSP-25, LMTA-34, LMTA-57, LMTA-94, and LMTA-148	Ready-to-eat foods: lettuce, 1.1 log_10_ reduction; cheese, 0.7 log_10_ reduction; smoked salmon, 1.0 log_10_ reduction; frozen entrées, 2.2 log_10_ reduction; apple slices, 1.1 log_10_ reduction after 24 h at 4 °C; elimination of detectable *L. monocytogenes* on naturally contaminated smoked salmon	[129]
ECP-100	STEC O157:H7	3 lytic Myoviridae phages: ECML-4, ECML-117, and ECML-134	Tomato, 94–99% reduction; spinach, 99–100% reduction; ground beef, approximately 95% reduction; hard surfaces, 85–100% reduction depending on phage titer and surface condition	[110]
SalmoFresh™	*S. typhimurium*, *S. heidelberg*, and *S. enteritidis*	Commercial lytic *Salmonella* phage preparation; phage cocktail applied as dip or surface treatment at 10^9^ PFU/mL	Chicken breast fillets: dip treatment reduced *Salmonella* by 0.7 and 0.9 log CFU/g on days 0 and 1 at 4 °C; surface treatment reduced counts by 0.8–1.0 log CFU/g under aerobic storage and by 1.1–1.2 log CFU/g under modified-atmosphere packaging	[130]
Broad-spectrum three-phage nontyphoidal *Salmonella* (NTS) cocktail	Nontyphoidal *S. enterica*, including *S. enteritidis*, *S. typhimurium*, and *S. kentucky*	3 broad-spectrum lytic phages	Raw chicken breast: >3.2 log_10_ reduction after 5 days at 10 °C; >1.7 log_10_ reduction after 16 h at 22 °C	[128]
Multireceptor five-phage cocktail	*S. enterica*	5 phage cocktail targeting O-antigen, BtuB, OmpC, and rough *Salmonella* phenotypes	Chicken skin: 3.5 log_10_ CFU/cm^2^ reduction after 48 h at 15 °C and 25 °C; 2.5 log_10_ reduction at 4 °C	[127]
Six-phage *Salmonella* cocktail/Applied Phage Meat S2	*S. enteritidis* and a five-serotype *Salmonella* mixture	6 phage cocktail; four myoviruses and two siphoviruses; applied by spray at 10^7^ PFU/cm^2^	Chicken skin: 1.8 log_10_ reduction for *S. enteritidis* and 1.0 log_10_ reduction for the five-serotype mixture after 30 min; up to 3.0 log_10_ after 4 h. Stainless steel: 1.2–1.7 log_10_ after 30 min and up to 2.4 log_10_ after 4 h; fresh wet contamination on stainless steel was reduced below detection after 2 h	[131]
Two-phage seafood cocktail	*V. parahaemolyticus*	vB_VpaS_1601 + vB_VpaP_1701	Salmon: 1.53–2.74 log CFU/cm^3^ reduction; oysters: 1.56–2.91 log CFU/cm^3^ reduction	[132]

* Note: “Below detection” indicates that bacterial counts were below the detection limit of the method used and does not necessarily indicate complete eradication. Abbreviations: CFU, colony-forming units; BtuB, vitamin B12 outer membrane transporter receptor; OmpC, outer membrane porin C; PFU, plaque-forming units; RTE, ready-to-eat.

**Table 6:** Representative engineered phage strategies with potential relevance to foodborne pathogen control.

Modification Type	Target Pathogen	Demonstrated Effect	Biosafety/Regulatory Status	References
Tail-fiber mutagenesis “phagebodies”	*E. coli*	Generated different phagebody libraries with ~10^7^-different members with altered host range; selected variants suppressed resistance over extended periods and remained active in a mouse wound model	Research-only platform; no food-use approval identified in official sources reviewed	[136]
Chimeric tailspike engineering	*S. enterica*	Expanded recognition across additional *Salmonella* serovars and improved specificity by eliminating off-target *Citrobacter* signal	Research/diagnostic prototype; no food-use approval identified	[137]
Tail-fiber engineering + CRISPR–Cas-armed lytic phage cocktail, SNIPR001	*E. coli*	Produced complementary CRISPR-armed phages with expanded receptor usage, reduced phage-tolerant emergence, biofilm activity, and improved gut decolonization in mice compared with individual components	Entered clinical development; medicinal route rather than food-use pathway	[138]
P1 phagemid delivery of CRISPR–Cas9	*Shigella flexneri*/*E. coli*	Achieved sequence-specific killing and reduced *S. flexneri* burden while improving host survival in zebrafish larvae	Research-only engineered delivery system; require additional safety evaluation before food-chain use	[139]
CRISPR/Cas9-engineered lytic phage displaying LL-37	*S.* Typhimurium	Enhanced antibacterial activity, prevented detectable phage resistance, reduced adhesion/invasion/intracellular burden, and improved *Galleria mellonella* survival	Early-stage experimental platform; no food-use clearance identified	[140]

## Data Availability

No new data were created or analyzed in this study. Data sharing is not applicable to this article.

## Abbreviations

The following abbreviations are used in this manuscript:

STEC	Shiga toxin-producing *E. coli*
AMR	Antimicrobial resistance
EU	European Union
FDA	Food and Drug Administration
GRAS	Generally recognized as safe
EFSA	European Food Safety Authority
USDA	United States Department of Agriculture
EPA	Environmental Protection Agency
FSANZ	Food Standards Australia New Zealand
HGT	Horizontal gene transfer
